# White blood cell differential fluorescence abnormal scattergram: A useful indicator for early detection of malarial parasite

**DOI:** 10.12669/pjms.38.3.4702

**Published:** 2022

**Authors:** Madeeha Rehan, Attika Khalid, Fariha Nasreen

**Affiliations:** 1 Dr. Madeeha Rehan, Associate Professor, Department of Pathology, Foundation University Medical College, Fauji Foundation Hospital Rawalpindi, Pakistan; 2 Dr. Attika Khalid, Assistant Professor, Department of Pathology, Foundation University Medical College, Fauji Foundation Hospital Rawalpindi, Pakistan; 3 Dr. Fariha Nasreen, Postgraduate trainee Haematology, Department of Pathology, Foundation University Medical College, Fauji Foundation Hospital Rawalpindi, Pakistan

**Keywords:** Malaria, Abnormal scattergram, Sysmex XN-1000

## Abstract

**Background & Objective:**

Undiagnosed malarial infectionis associated with significant mortality and morbidity. Laboratory investigations leading to rapid, accurate and timely diagnosis of malaria is still a challenge. This study was done to assess the utility of abnormal White blood cell differential fluorescence (WDF) scattergram for diagnosis of malaria. Our aim was to study the utility of WDF scattergram for early detection of malarial parasite.

**Methods:**

All EDTA anti-coagulated blood samples received in laboratory during a period from Dec 2019 to May 2020 were analyzed on anautomated hematology analyzer, Sysmex XN 1000. All abnormal WDF scattergrams pertaining to plasmodium specie were critically evaluated and recorded. Review of Leishman-stained peripheral smears as well as immune-chromatographic assay by rapid test devices (RTD) was done. Accordingly, sensitivity, specificity, positive predictive value (PPV) and negative predictive value (NPV) for detection of malaria by abnormal scattergram were calculated.

**Results:**

Out of total 1, 26,000of samples analyzed, abnormal WDF scattergrams were detected in 96 cases. Amongst these, 95.8% (92) were positive for MP on Leishman-stained peripheral smear as well as on ICT with a p-value of 0.05. WDF scattergram abnormalities typical of malaria showed a sensitivity of 80% and specificity of 93.26%. Positive predictive value of 95.8% whereas negative predictive value of 99.9% was detected. Significant findings of hemolysis, platelet clumps, nucleated RBCS (NRBCs) and RBC agglutination were noted in cases (n=4) with abnormal WDF scattergram negative for malaria on peripheral smear.

**Conclusion:**

Interpretation of abnormal WDF scattergram not only increases the early detection rate for malarial parasite but isa strong indicator for presence of hemolysis, RBC agglutination, platelet clumps and leucoerythroblastic blood picture as well.

## INTRODUCTION

Despite advances in diagnosis and management of malaria over the past decade, it remains the most common and important human vector-borne disease worldwide. Amongst the four known species of plasmodium (P) globally, the dominant species is P. falciparum however *P.vivax* is the most commonly found species in Afghanistan, Pakistan, Iran and Iraq. In Pakistan, 1.5 million cases per annum have been estimated. In different provinces of Pakistan, the prevalence of *P.vivax* found to be 2.4% in Punjab, 10.8% in Sindh whereas prevalence of *P.falciparum* ranged from 0.1% in Islamabad and 3.8% in Balochistan.^1^

Microscopy of Giemsa-stained peripheral smears of patients with suspected malaria are still gold standard for its diagnosis. The liability of this method is based on the professional expertise in staining technique and microscopy. Moreover, it is also time consuming in setups with heavy workload and limited resources. Keeping these limitations in view, several new techniques for detection of malarial parasite were offered. These include antigen-coated dipstick tests, quantitative buffy coat examination using fluorescent dyes and polymerase chain reaction.^2^,^3^ Although these tests are sensitive and specific in malaria detection but are expensive and have less availability.^4^ Sensitivities of different immunologic methods for detection of malarial parasite is still a problem in detection of lower level of parasitaemia.^5^

Several latest automated haematology analyzers indicate flagging or graphical representation of malaria as a new emerging technique for its early detection. Automated haematology analyzers e.g., Sysmex XN-1000 perform a complete blood count and detect the presence of plasmodium species by showing an abnormality of the WDF, WNR, and RET scattergram. WDF scattergrams are graphical representation of the findings of the WDF channel. Different types of white blood cells are counted in WDF channel in XN series.^6^ It is based on principle of flowcytometry using a semiconductor laser to produce three types of optical data about different cells. Forward scatter light (FSL) as a cell size indicator, side scatter (SSC) as an indicator of the complexity of internal structure such as granules and nuclear content is indicated by side fluorescence light (SFL). Hemozoin, which is produced during hemoglobin catabolism, is phagocytized by neutrophils and monocytes leading to generation of abnormal scattergrams thus leading to the identification of malarial infection by automated methods.

Various studies have been conducted worldwide based on results of the automated hematology analyzers like, GEN S andLH750, XE2100 and XS100i7 for malarial detection.^7^ This study is an attempt to highlight the significance of WDF scattergram interpretation for early detection of malaria in unsuspected cases of malaria. It will not only be useful in underdeveloped countries, laboratories with increased workload and limited resources especially in endemic regions but also alarming for the pathologist to examine the peripheral blood smears more vigilantly thus preventing misdiagnosis and planning of timely therapeutic intervention for prevention of disease complications.

## METHODS

It was a prospective study conducted at Fauji Foundation Hospital Rawalpindi over a period of six months duration from December 2019 to May 2020. After taking ethical approval from ethical review board (Ref. No. 410/ERC/FFH/Rwp, dated: 11/08/2020), all the complete blood counts (CBC) samples received in pathology laboratory from both outpatient departments (OPD) as well as wards were processed on automated Haematology analyzer Sysmex XN-1000. All the CBCs that were requested specifically for malaria and ICT MP were excluded from the studies. The CBCs showing abnormal scattergram pattern pertaining to malaria were selected and Leishman-stained peripheral smear stained were prepared and examined. ICT MP was also performed for all these selected cases. The abnormalities in the scattergram included double neutrophil line, multiple neutrophil lines, pseudo eosinophilia, right ward shift of ghost area and greying of the neutrophil/eosinophil zone. The peripheral smear was reviewed by post graduate resident and verified by consultant haematologist. All cases found to be positive for plasmodium species were documented in the software system and notified to the respective wards. The walk-in patients and patients from OPD were contacted on cell numbers to report to hospital immediately. Data analysis was done through SPSS version 17. All quantitative variables like age and gender were noted. Frequency and positive predictive and negative predictive value of malaria positive cases with abnormal scattergram was recorded. Sensitivity and specificity were also calculated for these selected cases.

## RESULTS

A total of 1,26,000 CBCs received in hematology laboratory during the period of six months (Dec 2019-May 2020) were analyzed along with their respective WDF scattergrams. Of the total CBC, 75,600 received were of admitted cases in various wards, 44,100 cases were booked from outpatient department and6,300 CBC were booked directly in the laboratory as walk-in patients. Amongst the total cases, 96 CBC cases showed abnormal WDF scattergram pertaining to malaria. Of these 96 cases, 92 cases were positive for malarial parasite on peripheral smear and ICT with a p-value of 0.05. 79.3% (73) were females and 20.6% (19) were males with mean age of 40 years.

Amongst these, 93.7% (90) were found to be positive for MP on Leishman-stained peripheral smear as well as on ICT MP. While 02 cases with no evidence of malaria on peripheral smear tested positive on ICT.

Out of the total 96 cases showing abnormal scatter gram, 4.1% (4) cases tested negative for malaria species on both peripheral smear as well as on ICT showed other findings such as RBC agglutination, platelet clumps, leucoerythroblastic blood picture and hemolysis on peripheral film examination (Table-II).

Amongst these cases only one case had plasmodium falciparum whereas mixed infection with plasmodium vivax and falciparum was detected in two cases. The rest of the 89 cases showed plasmodium vivax. No Ovale, Malariae species were reported (Table-I). Sensitivity, specificity, positive and negative predicted value were calculated (Table-III).

**Table I:** Frequency of plasmodium specie observed in positive cases.

S. No.	Plasmodium specie	Number of Cases
1	Plasmodium Vivax	89
2	Plasmodium Falciparum	01
3	Mixed infection with plasmodium Falciparum and Vivax	02
4	Plasmodium Malariae	Nil
5	Plasmodium Ovale	Nil
6	Total	92

**Table II:** Findings of cases with abnormal scatter gram but negative ICT MP and peripheral smear.

Findings of cases with abnormal scatter gram but negative ICT MP and peripheral smear	Number of cases (%)	Observation
Agglutination	2	Peripheral smear
Hemolysis	1	Gross examination and peripheral smear
Thrombocytosis and nucleated RBC	1	Peripheral smear

**Table III:** Positive and negative predictive value, sensitivity and specificity.

Positive and negative predictive value Sensitivity & specificity	Frequency
Positive predictive value	95.8%
Negative predictive value	96.8%
Sensitivity	96.8%
Specificity	99.9%

## DISCUSSION

The detection of clinically unsuspected malaria is of significant importance in order to prevent the mortality and morbidity of the disease.^8^ There is usually limited access to clinical and laboratory expertise for identification, detection, and quantification of malaria in most endemic areas. Fluorescent and immunological tests are prohibitively expensive.^4^ In non-endemic countries, a differential diagnosis of malaria is usually not considered in acute febrile illness by most of the clinicians.^5^ In unsuspected patients, early detection of malaria is an emerging screening tool by modern haematology analyzers.

In various studies Haematology analyzers evaluated for malaria detection include the Cell-Dyn (Abbott Diagnostics, Santa Clara, CA),3 the GEN.S and LH-750 (Beckman Coulter, Miami, FL),4 and the XE-2100 and XS-1000*i* (Sysmex Corporation, Kobe, Kansai, Japan).^6^-^8^ Principle of flowcytometry is used in automated haematology analyzers and semiconductor laser is used to generate three types of optical data about cells.^9^ In this study a fundamental importance has been given to the malarial parasite detection by WDF abnormal scattergram (graphical representation of the findings of the WDF) generated by automated hematology analyzers XN-1000 for early detection of malaria even in absence of a specific clinical request. White cell differential channels in XN series is channel for counting the number of different white cell types present in the blood sample. In the DIFF channel (differential count) surfactant is used to lyse red blood cells and platelets and a polymethine dye to bind to nucleic acid which gives fluorescence signal intensity proportional to nucleic acid content. Furthermore, it uses an organic acid which specifically bind to eosinophil granules to be differentiated from neutrophils by high side scatter intensities.^10^,^11^

The present study showed abnormalities in hematological parameters like anemia, leukopenia and thrombocytopenia which were also reported in previous studies.^12^ Abnormal WDF scatter grams were detected in 96 cases. Out of these 96 cases, 90 were positive on peripheral smear as well as on ICT for MP. Two cases negative on peripheral smear were found to be positive on ICT.

A study conducted in India by Ningombam A et al showed 99.2% parasite detection by abnormal scattergram on similar haematology analyzer used in present study thus showing comparable results.^13^

In present study 89 cases were positive for plasmodium vivax, two for falciparum and one was mixed infection. It is in accordance with most of the studies reported plasmodium vivax as the most common infection globally. *P.vivax* is the most common plasmodium infections in Pakistan but P. falciparum and mixed species infections are also prevalent.^1^ In the present study, the sensitivity was 96.8% and specificity was found to be 99.9%. This is in accordance with a study conducted by Pilley et al., which suggested that there was correlation between microscopy and the abnormal scatter-grams generated by haematology analyzer and reported 100% sensitivity and specificity. However in contrast to our study the results were generated on XN-30.^14^ Another study by Sharma et al. suggested a sensitivity of 83.78% and specificity of 94.82%.^2^ In a study conducted in South Korea on XE 2100 analyzer showed comparable results to our study in which Yoo et al. reported sensitivity and specificity of 46.20% and 99.70% respectively. However, the main focus in their study has been only pseudo eosinophilia as a predictive marker for malaria detection whereas in our study we included all abnormal patterns observed in WDF channel.^15^Hänscheid et al. concluded in their study that in comparison to microscopy, scatter flow cytometry using Cell-Dyn 3000 instrument detected significantly more patients with malaria.^16^

A study conducted by Zuluaga GC performed on XE-2100 indicated that malaria alarm produced by abnormal WDF scattergram can be helpful for early malaria microscopic diagnosis.^17^

According to the study conducted by Buoro et al. nine patients were positive to Plasmodium falciparum, one to Plasmodium ovale, and one to Plasmodium malaria and three to Plasmodium vivax. Significant abnormalities were observed in both white blood cell differential (WDF) and white cell nucleated (WNR) scatter grams (sensitivity 0.64 and specificity 1.0) in nine samples with parasites at gametocyte or schizonts stage, while characteristic scattergram abnormalities were not detected in the 5 samples containing only parasites at the trophozoites stage. The specific variations of some cell population data (CPD) could be recorded (sensitivity 1.00 and specificity 0.91) in these samples.^18^

In another study of the 80 patients who presented with fever and suspicion of malaria, 29 patients were positive for malaria and 10 cases were diagnosed incidentally by the findings on the cell counter and were confirmed by Giemsa-stained blood smears. The sensitivity and specificity of the abnormalities detected in the WBC-Diff channel in detecting malaria is 82% and 100% respectively. Using WBC-BASO channel abnormality for initial diagnosis the sensitivity and specificity is 50% and 92.5% respectively.^19^ According to a study by Pillai KR et al., P.vivax was the most dominant species (60.8%). Greying of the both neutrophil and eosinophil populations was seen in 75% of vivax (p-value=0.019) and 23.2% of falciparum cases.^20^ In present study positive predictive value of 95.8% whereas negative predictive value of 99.9% was calculated. It reflected similarity with the results of study conducted by Kumar S & Naik P which showed a sensitivity of 80% and specificity of 93.26% whereas positive predictive value was found to be of 40% and a negative predictive value of 98.81%.^6^ The cases having abnormal scatter grams but negative for malarial parasite on both smear and ICT found to have evidence of hemolysis, RBC agglutinates NRBCs and leucoerythroblastic blood picture on peripheral smears thus indicating other underlying clinical disorders. Platelet clumps were also identified in few of these cases. Although it is evident from literature that almost 10% non-malaria cases with different underlying clinical conditions e.g., thalassemia, leukemia, hemolytic diseases of newborn and septicemia showed various abnormalities in WDF scattergram mimicking malaria^3^ but there is scarcity in literature in this context. However, these findings are of significant importance for diagnosis of the serious underlying conditions and should not be overlooked. In another study conducted by Campuzano-zuluaga et al similar findings were described using *P.vivax* infected samples, however in contrast to our study an increase in highly fluorescent lymphocyte-coded along with elevated reticulocyte/IRF ratio events in the DIFF scatterplot in a *P. falciparum*-infected patients were also detected.^7^ A study conducted in Pakistan concluded that in endemic areas with unavailability of expert microscopists automated haematology analyzers can be used as a useful adjunct for timely clinical diagnosis of malaria.^21^

### Limitations of the study

It is a single center study. This study should be extended on multicenter to better enhance and validate the research findings. Furthermore, this idea can also be replicated for different CBC analyzers to assess their utility in this regard as well.

## CONCLUSION

Timely detection of malaria is life-saving. Knowledge about interpretation of abnormal WDF scattergram generated by automated haematology analyzers and its application can be utilized as an adjunctive diagnostic tool for early diagnosis of malaria especially in unsuspected cases where diagnosis can be overlooked. It has the potential to facilitate the diagnostic approaches, and significantly impact therapeutic monitoring and efficacy of anti-malarial and even clinical trials. It can also serve as the ideal blood donor screening tool for presence of malaria especially in malaria-endemic regions.

### Authors’ Contribution:

**MR:** Literature search, manuscript writing, data acquisition, manuscript drafting and editing and critical review for intellectual content and final approval of the version to be published. Corresponding author and accountable for accuracy and integrity of work.

**AK:** Concept and design of the study, data acquisition and statistical analysis, critical review.

**FN:** Data collection, Data analysis & Data interpretation, critical review.
